# Systematic review of Alzheimer's Disease (AD) biomarker studies in those with intellectual and developmental disability (I/DD): Evidence gap mapping

**DOI:** 10.1002/alz70856_106296

**Published:** 2026-01-09

**Authors:** Lubnaa B Abdullah, Ney Alexander Alliey, Emma Elizondo, Ney Alliey‐Rodriguez, Gladys E. Maestre, James Hall

**Affiliations:** ^1^ University of North Texas Health Science Center, Fort Worth, TX, USA; ^2^ University of Texas Rio Grande Valley, Edinburg, TX, USA; ^3^ Institute of Neuroscience at the University of Texas Rio Grande Valley, Harlingen, TX, USA; ^4^ The University of Texas Rio Grande Valley School of Medicine, Harlingen, TX, USA; ^5^ Institute for Translational Research, University of North Texas Health Science Center, Fort Worth, TX, USA

## Abstract

**Background:**

The relatively small number of investigations into brain aging in those with intellectual and developmental disabilities (I/DD) has been restricted to homogenous samples with Down Syndrome (DS), resulting in a significant gap in scientific information available. Objectives: To a) characterize the internationally available multi‐omics AD biomarker studies in those with I/DD, b) provide an overview of the current evidence gaps and c) discuss implications and future research directions aimed at filling those gaps.

**Method:**

PubMed, Web of Science, Scopus were searched for key terms under the following criteria: cross‐sectional or longitudinal AD‐omics studies on adults (18 +) with I/DD.

**Results:**

532 studies were identified. 186 studies were evaluated for full text. 79 studies were excluded; 117 studies were extracted.

**Discussion:**

Study populations were primarily of European ancestry from the United States, United Kingdom, and Spain. There were only 3 studies from Japan while there were no studies from Africa, Oceana, or South America represented. Most biological specimens were analyzed in blood plasma or serum. Three studies analyzed biospecimen in saliva and 1 study used retinal tissue. Metabolomics, hormonomics, and transcriptomics was most understudied. 17 and 8 studies considered comorbidities or social determinants of health, respectively. Sex differences were investigated in 9 studies. Two studies included participants with non‐DS neurodevelopmental disorders.

**Limitations:**

This study excluded post‐mortem/brain sample studios, thereby restricting exploration into transcriptomics.

**Conclusion:**

The lack of data from representative samples of individuals with I/DD limits the generalizability of current study outcomes. Future studies should include a broader range of I/DD presentations. In addition to non‐DS I/DD presentations, sex differences and novel biomarkers such as beta synuclein and VAMP‐2 are promising future directions.

